# Spatiotemporal Eye-Tracking Feature Set for Improved Recognition of Dyslexic Reading Patterns in Children

**DOI:** 10.3390/s22134900

**Published:** 2022-06-29

**Authors:** Ivan Vajs, Vanja Ković, Tamara Papić, Andrej M. Savić, Milica M. Janković

**Affiliations:** 1 School of Electrical Engineering, University of Belgrade, Bulevar Kralja Aleksandra 73, 11120 Belgrade, Serbia; andrej_savic@etf.rs (A.M.S.); piperski@etf.rs (M.M.J.); 2 Innovation Center, School of Electrical Engineering in Belgrade, Bulevar Kralja Aleksandra 73, 11120 Belgrade, Serbia; 3 Faculty of Philosophy, University of Belgrade, Čika-Ljubina 18-20, 11000 Belgrade, Serbia; vanja.kovic@f.bg.ac.rs; 4 Faculty of Technical Sciences, University Singidunum, Danijelova 32, 11000 Belgrade, Serbia; tpapic@singidunum.ac.rs

**Keywords:** developmental dyslexia, reading, screening, colored background, eye-tracking, feature extraction, machine learning, support vector machine, k-nearest neighbors, random forest, logistic regression

## Abstract

Considering the detrimental effects of dyslexia on academic performance and its common occurrence, developing tools for dyslexia detection, monitoring, and treatment poses a task of significant priority. The research performed in this paper was focused on detecting and analyzing dyslexic tendencies in Serbian children based on eye-tracking measures. The group of 30 children (ages 7–13, 15 dyslexic and 15 non-dyslexic) read 13 different text segments on 13 different color configurations. For each text segment, the corresponding eye-tracking trail was recorded and then processed offline and represented by nine conventional features and five newly proposed features. The features were used for dyslexia recognition using several machine learning algorithms: logistic regression, support vector machine, k-nearest neighbor, and random forest. The highest accuracy of 94% was achieved using all the implemented features and leave-one-out subject cross-validation. Afterwards, the most important features for dyslexia detection (representing the complexity of fixation gaze) were used in a statistical analysis of the individual color effects on dyslexic tendencies within the dyslexic group. The statistical analysis has shown that the influence of color has high inter-subject variability. This paper is the first to introduce features that provide clear separability between a dyslexic and control group in the Serbian language (a language with a shallow orthographic system). Furthermore, the proposed features could be used for diagnosing and tracking dyslexia as biomarkers for objective quantification.

## 1. Introduction

Individual differences in learning to read originate from biological and environmental factors, which shape the development of the brain systems involved in the reading process [1]. Dyslexia, a specific learning disorder with impairments in reading [2] refers to a pattern of learning difficulties characterized by problems with accurate or fluent word recognition, poor decoding, and poor spelling abilities. Up to 20% of the general population may exhibit some degree of these difficulties [3], while about 7% of people are affected heavily enough to qualify for a dyslexia diagnosis [4]. Due to its nature, dyslexia is typically diagnosed only after children have started to learn to read, when it becomes evident that they are struggling to keep up with their peers [5]. At this point, the pupils with dyslexia are already at risk of falling behind because reading is essential for school achievement in most subjects. Moreover, children with poor reading skills are also at an increased risk of social, emotional, and mental health problems, such as school dropout, attempted suicide, incarceration, anxiety, depression, and low self-concept [6]. Therefore, it would be invaluable if children with dyslexia or at risk for dyslexia could be identified and involved in prevention and treatment programs as early as possible.

The diagnosis of dyslexia, especially at its early stages, has proven to be a complex task, especially because of the lack of a strict procedure for dyslexia screening [7]. Being able to diagnose dyslexia and create a tool that can objectively quantify certain dyslexic tendencies has proven to be quite important so that the diagnosis process could be as objective and reliable as possible [8].

Recently, Carioti et al. have shown that in developmental dyslexia research (published from 2013 to 2018), 67.4% of studies were performed on languages that could be considered to have a deep orthographic system [9]. Considering this, performing dyslexia research on languages that have a shallow orthographic system could be considered quite important, not only because of their underrepresentation but because reading in a shallow orthographic system is easier, making dyslexia even harder to diagnose. Dyslexia diagnosis is a challenging task in the Serbian language (with one-to-one grapheme-phoneme pairs), which belongs to the group of languages with a shallow orthography.

In this paper, the study performed on native dyslexic and non-dyslexic Serbian speakers is presented. Novel spatiotemporal eye-tracking features were introduced, and the classification results using various machine learning (ML) algorithms were compared with the results obtained using conventional eye-tracking features. The difference between the subject classes (dyslexic and non-dyslexic) was analyzed using statistical tests for different color configurations in order to examine the influence of the color configuration of the reading material on subject class separability. Statistical analysis was also performed within the dyslexic subject group in order to analyze the influence of color configuration on reading performance and to determine whether a given color could influence the eye movement features in a manner indicating facilitation or aggravation of the reading task in subjects with dyslexia.

The contributions of the performed research are as follows:

- Development of a novel feature set for describing and quantifying dyslexic tendencies in the Serbian language;
- Statistical and classification analysis, showing the potential of the proposed features to be used as indicators of dyslexic tendencies;
- An analysis of the influence of colored backgrounds and overlays on reading patterns using a selection of the proposed features that have shown to be the most indicative of dyslexic reading patterns.

## 2. Related Work

Dyslexia is diagnosed by tests that include reading and writing assessments, among other evaluations, and are standardized by experts on a large number of subjects [10,11]. The advancement of technology has made the digitalization of these tests possible, and it has also contributed to the objectivity of the testing as certain quantifiable metrics can be obtained from digitalized dyslexia tests [12,13,14].

Different screening methodologies can be performed to distinguish dyslexic from non-dyslexic subjects. Brain-imaging methodology most prominently focuses on functional magnetic resonance imaging during reading [15,16] and diffusion tensor imaging [16,17], which both show, respectively, the functional or morphological differences between the dyslexic and the control group. Brain activity can be monitored using electroencephalography (EEG) as well, either on its own [18,19,20] or in combination with other biometric signals, such as heart rate, electrodermal activity (EDA), and eye tracking [21,22,23,24].

The analysis of reading and eye movement patterns is often performed in dyslexia research. Temelturk et al. in [25] performed a systematic review of 25 papers that include binocular eye-tracking during linguistic and non-linguistic tasks in children from 5–17 years of age with dyslexia and with typical development. The review aimed to combine the knowledge from the existing literature that observed the binocular coordination in children with dyslexia by describing the normative development of stable binocular control. The findings of the review indicate clearly that there is poor binocular coordination in children with dyslexia but that the results associated with different task characteristics were not as consistent. Another study focused on detecting dyslexia based on reading patterns was presented by Wang et al. in [26]. A neural network was developed that was used to predict whether or not the subject had developmental dyslexia, based on the data gathered from 399 Chinese children. The dataset included children aged 7–13, 187 with dyslexia and 212 controls. The authors report an achieved accuracy of 94%, claiming that the reading accuracy was the feature that had the strongest factor in detecting dyslexia, but the phonological awareness, the accuracy rate of pseudo characters, the morphological awareness, the reading fluency, the rapid digit naming, and the reaction times of noncharacters made important contributions to the classification as well.

Eye tracking is often used in the practical diagnosis of dyslexia as it provides a direct insight into the visual sampling strategy. The eye movements of subjects with dyslexia show an erratic gaze pattern that can be quantitatively described by features and used for further development of the algorithms for automatic dyslexia recognition [27].

Rello et al. in [28] claim to be the first to attempt classifying dyslexia based on eye-tracking features using machine learning. The language of the text used in the experiment was Spanish, and 97 subjects were included (48 with dyslexia), with the subject age ranging from 11 to 54. Each subject read 12 different texts, each presented in a different font type, on white paper with black letters. A support vector machine (SVM) classifier was implemented, and the features used as inputs were the age of the participant, mean duration and the total number of fixations, total reading time, etc. The model was evaluated using 10-fold cross-validation, and an accuracy of 80.18% was achieved.

A study with a larger number of participants and a more in-depth feature analysis was performed in [29]. The data were gathered from 185 subjects (97 with dyslexia), with ages ranging from 9 to 10, who read a single text written in the Swedish language. The text was presented on white paper with black letters, and a total of 168 eye-tracking features were considered. The features were derived from both version and vergence [30], the regressive and progressive movements, the saccades, the fixations, the duration of the event, the distance spanning the event, the accumulated distance of an event, the accumulated distance over all subsequent positions, etc. Considering the large number of features, a recursive feature elimination (RFE) algorithm was implemented to reduce the number of features. An SVM classifier was used, and it was evaluated using 10-fold cross-validation, which was repeated 100 times to ensure the stability of results in terms of dataset splitting. The highest achieved accuracy was 95.6%, and it indicates that a large number of subjects in combination with a wide range of observed features enables a reliable classification. This paper also effectively performed subject-wise evaluation, where the data from a given subject are either in the training or test, creating an evaluation scenario similar to a real use case [31].

Prabha et al. [32] analyzed the dataset introduced in [29] using several ML algorithms. Only the features extracted from fixations, in combination with an RFE feature selection algorithm, were used for the classification. The authors implemented an SVM classifier (with four different kernel configurations), a k-nearest neighbors (KNN), and a random forest (RF) algorithm and achieved the highest accuracy of 95% by KNN. In their further work [33], Prabha et al. focused on analyzing the same dataset, but with new ML algorithms, such as particle swarm optimization (PSO)-based SVM hybrid kernel (hybrid SVM–PSO), SVM, RF, logistic regression (LR), and KNN. They also observed features extracted from both saccades and fixations and obtained an accuracy of 95.6% with the hybrid SVM–PSO model. Prabha et al. also focused on observing eye-tracking feature sets and several other ML algorithms in their work performed on the same dataset [34,35], obtaining similar results, although a slightly higher accuracy of 96% in [35] using a hybrid SVM–PSO model.

A study including 69 children (32 with dyslexia) was conducted in [36]. The children were aged 8.5–12.5 and read two text paragraphs in Greek. The authors implemented several ML algorithms (KNN, SVM, and naïve Bayes) and observed a wide range of eye-tracking features. The best-obtained accuracy of 97% was achieved using only three features, saccade length, the number of short forward movements, and the number of repeatedly fixated words.

A holistic approach for dyslexia detection based on a convolutional neural network (CNN) was implemented in [37]. The authors used the dataset from [29], but rather than extracting features, they used gaze coordinate data as a direct input to the CNN and implemented several padding algorithms to make the data sequences the same length. The achieved accuracy results of 96.6% (obtained with a modified cross-validation evaluation) show that, given the right data encoding, deep learning algorithms can provide very reliable dyslexia detection based on eye movement data.

Weiss et al. [38] analyzed the lateralization of early orthographic processing during natural reading in subjects with dyslexia. The authors recorded the eye-tracking and EEG activity of the subjects, 24 subjects with dyslexia (mean age 24.8) and 24 control subjects (mean age 23), during the reading of isolated sentences in their native (Hungarian) language, with various spacing between letters. The statistical analysis of the EEG and the eye-tracking parameters performed in the paper has shown several interesting findings. Increased spacing between letters was shown to reduce the silent reading speed in both subject groups, in contrast to the beneficial effects on oral reading found in previous work. Furthermore, the authors found that the early left-hemispheric lateralization of orthographic processing during natural reading depends on the rank of fixations and that it is most prominent when reading on the default letter spacing in control readers, as well as that it deteriorates in subjects with dyslexia.

The detection of developmental dyslexia using machine learning and eye movement data was performed in [39]. The authors observed a group of 165 subjects with an average age of 12.5. Of the chosen subjects, 30 met the criteria for a reading disorder based on choosing the 10th worst percentile of the reading fluency performance score, which was used to label them as dyslexic. The language used in the reading experiment was Finnish (the subjects’ native language), and a variety of eye-tracking features were observed. An RF algorithm was used for feature ranking, and an SVM was used for subject classification based on the selected features. The overall accuracy of 89.7% was achieved using five-fold cross-validation.

El Hmimdi et al. [40] performed research on predicting a dyslexia diagnosis as well as reading speed from eye movement data in both reading and non-reading tasks. The authors used eye movement measures from four different setups, gathered from 46 dyslexic subjects (average age 15.5) and 41 control subjects (average age 14.8), recruited from schools in Paris. A vergence, saccade, and two reading tests were performed by each subject, and several eye-tracking measures were derived from the obtained data. Based on the obtained features, a variety of ML algorithms were implemented, and the findings showed an accuracy of 81.25% percent when using the data from the reading tests and 81.25% and 77.3% accuracy from the two no-reading tests, respectively. The prediction of reading speed was also performed on each of the feature sets from the two reading tests and two no-reading tests, showing that the reading speed can be predicted more accurately from one non-reading task than from the two reading tasks.

Vajs et al. [41] presented a CNN solution for dyslexia detection based on the VGG16 neural network architecture. The eye-tracking data were gathered from 30 subjects (ages ranging from 7–13), 15 with dyslexia, and 15 controls. The subjects read the text in their native language (Serbian) on different colored backgrounds and overlays, and the raw eye-tracking data were segmented, visualized, and used in the form of colored images as inputs to the CNN model. The model was evaluated using leave-one-out subject cross-validation, and an accuracy of 87% was achieved.

## 3. Materials and Methods

### 3.1. Dataset and Experiment Description

The data analysis in this paper was conducted on the dataset described in our previous research [22,41]. The data were gathered from 30 subjects, 15 diagnosed with dyslexia and 15 control subjects (age: 7–13, gender: 19 female, 11 male), during a study approved by the ethical committee of the Psychology Department of the University of Niš (a branch of the Serbian Psychology Association), experimental procedure No. 9/2019. The subjects could withdraw from the test at any time. In consultation with a certified speech therapist, the subjects with dyslexia were selected from several elementary schools in Belgrade. The control subjects were selected randomly from three elementary schools in Belgrade. The age range in the group with dyslexia was 7–13 years, of which 4 were male and 11 were female, with an average age 9.93. The age range in the group without dyslexia was 7–13 years, of which 7 were male and 8 were female, with an average age 9.67. All of the children in the sample, both dyslexic and non-dyslexic, had normal (or corrected to normal) vision. The children did the study in the morning hours during the regular school schedules.

During the experiment, the children were alone in an isolated, quiet, and bright room with the experimenter, sitting on a chair at a table in front of a computer monitor and keyboard. The screen size was 48 cm × 27 cm, the brightness was set to 90%, the distance from the screen was 62 cm; this was the same for all the participants. Additionally, we used the chin-rest so that the position of the head/eyes relative to the monitor was the same. During the experiment, each subject read 13 segments of the text extracted from a standardized story for elementary school called “Saint Sava and the villager without happiness”. At the beginning of the experiment, the subjects were instructed to read the text quietly for themselves from the stimuli presentation shown and to press the space button for the next slide of the stimuli presentation. The experiment was run applying the pseudo randomization of color background/overlay order, always starting with a referent slide (black text on white background). No other color was fixed/related to a certain text. Therefore, in this way, any other factors apart from the actual color would be averaged out (paragraph complexity such as vocabulary, syntax, etc., as well as semantic/affective content). The text was prepared and presented within the SMI Experiment Center software 3.7, keeping the same size/font for each slide, centrally presented with approximately the same length. All the colors (color shades) used for designing the slides (stimuli) were defined within the RGB color model, and each individual color was expressed as an RGB triplet ([R,G,B]), where the value of each additive primary color component can vary from 0 to 255. A list of background shades in the slides with colored backgrounds (and black text) with the associated numerical values of their RGB triplet is stated in Section 2.3 Experiment Design of our previous study [21]. An example of the test boards used in the experiment is attached in the Supplementary Materials. The reading of each text segment, for one subject, will be called “a trial” in the further text, although 30 subjects were included in this study, each with 13 trials. The trials with insufficient focus on the displayed text (reading time less than 5 s) were excluded, resulting in a total of 378 trials used for further analysis.

Several biometric parameters of the subjects were monitored during the reading task using a multimodal sensor hub [21]. The hub performed heart rate monitoring, EEG, EDA monitoring, and eye tracking. This study, however, was focused on the eye-tracking aspect of dyslexia, recorded by an SMI RED-m 120 Hz portable remote eye tracker (iMotions, Copenhagen, Denmark). Eye-tracker calibration was conducted in the SMI BeGaze software 3.7 (SensoMotoric Instruments, Teltow, Germany), and the experiment could be initiated only if the calibration had been successfully conducted. The acceptable accuracy for the 5-point calibration and validation was 0.5 degrees for both axes. Data validation in the form of the visual inspection was performed immediately after each recording session, using the BeGaze software. Each trial was adequately characterized by 3 data sequences, one representing the x coordinates, the other representing the y coordinates of the gaze, and the final one representing the event status signal of the recording (indicating the following events: fixation, saccade, blink/missing data).

### 3.2. Data Visualization and Feature Extraction

An original visualization technique was implemented so that the gaze data could be easier to analyze and display in a more intuitive manner. The x and y  gaze coordinates of a given trail are plotted in an x−y plane, following several rules:

- the color of the gaze line plotted between points $p_{k-1}=(x_{k-1},y_{k-1})$ and $p_{k}=\left(x_{k},y_{k}\right)$ is calculated based on the distance between points $p_{k-1}$ and $p_{k}$, using a jet color map (color map covers the line length range from 0 to 200 pixels, where 200 pixels is the maximal length of a saccade in the experiment, excluding saccades that occur between two lines of text);
- lines that connect the gaze points that belong to fixations are connected fully, while the lines that connect the points belonging to saccades are dashed;
- the last recorded gaze coordinates before and after a detected blink state are marked with red stars;
- the opacity of the line connecting the gaze points decreases over the course of the trial (time t) according to the following equation
  (1) $${\mathrm{opacity}}_{t}=0.9-0.8*\min\left(1,\frac{t}{MRT\times T_{s}}\right),$$
- where $T_{s}$ represents the sampling frequency ($T_{s}=60\;\mathrm{Hz}),$ and the opacity ranged from 1 (completely opaque) to 0 (completely transparent). The opacity is calculated so that it linearly decreases over time, up to the MRT, which represents the maximum reading time in this study (40 s).

**Figure 1:**
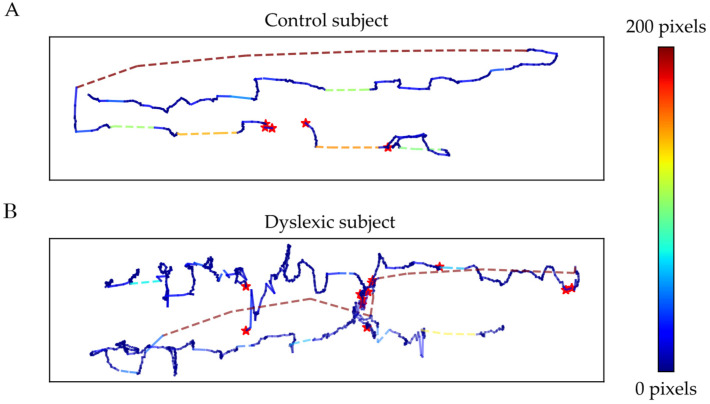
Trial visualization example for (**A**) a control subject and (**B**) a dyslexic subject. The color of the line represents the line length in pixels according to the presented color scale and the red stars represent blink events.

An example of trial visualization is given in Figure 1.

By analyzing the visualized trials, the global tendencies of the dyslexic subjects could be observed, which were then quantified by signal features.

The nine conventional eye-tracking metrics used as features for classification were: *Fixation count, Saccade count*, *Fixation frequency*, *Saccade frequency*, *Fixation average duration*, *Saccade average duration*, *Fixation total duration*, *Saccade total duration*, and *Total reading time* [42].

Aside from the commonly analyzed eye-tracking metrics, three new spatial features were introduced, as well as two new temporal ones. The three spatial features are related to fixation events and do not directly rely on the fixation duration or number of fixations. Rather, they are focused on quantifying the irregularity and complexity of the gaze during fixation events in the x−y coordinate plane. The first proposed spatial feature is called the *Fixation intersection coefficient* (FIC), and it is calculated per trial as

(2) $$FIC=\frac{1}{n}\overset{n}{\underset{j=1}{\sum }}FI_{j}$$

where n represents the number of fixations in a trial, and $FI_{j}$ represents the number of self-intersections of the lines belonging to the fixation j in the x−y plane. This feature was introduced because a higher number of self-intersections of gaze lines during fixations was observed in dyslexic subjects when compared to the control ones. The second spatial feature metric is called *Fixation intersection variability*, and it represents the standard deviation of the previously described $FI_{j}$ array. This was introduced because the number of self-intersections in fixation gaze lines varied more within a single trial for dyslexic subjects when compared to the control subjects. The third spatial feature is called the *Fixation fractal dimension* (FFD), and it is calculated per trial as

(3) $$FFD=\frac{1}{n}\overset{n}{\underset{j=1}{\sum }}FD_{j}$$

where $FD_{j}$ represents the fractal dimension of the figure created by the lines belonging to the fixation j in the x−y plane, estimated by the box-counting method [43]. This feature was introduced to directly quantify the complexity of the fixation gaze lines.

In addition to the spatial features, two temporal features were introduced, named *Active reading time* and *Saccade variability*. The *Active reading time* is calculated as the time spent in the fixation and saccade states, effectively excluding the time spent in the blink state or the intervals where the gaze was not detected. It was introduced with the goal of observing only the time spent actively reading the displayed text. Finally, *Saccade variability* was calculated as the standard deviation of the time intervals between two succeeding saccades. This feature was introduced by focusing on the observed tendency of the saccadic events to be more equally spaced out in the control subjects, as opposed to the dyslexic ones.

The data visualization and feature extraction were implemented in the Python 3.8.1 environment.

### 3.3. Machine Learning and Statistical Analysis

After the feature extraction, each trial was represented by a set of 14 (9 conventional and 5 proposed) features and its appropriate label (control or dyslexic). The obtained dataset was used to train four ML algorithms as well as to perform statistical analysis.

The selected ML algorithms were the LR, SVM, KNN, and RF. They are implemented in the Python 3.8.1 programming language, using the sklearn library [44]. When training each of the algorithms, the training set was standardized (made to have a mean value of 0 and standard deviation of 1), and the parameters for standardization on the train set were later used on the test set. The training/hyper parameters of the models were kept at their default values from the sklearn library (aside from the probability parameter used in the SVM implementation) and are as follows for the used ML algorithms:

- LR: penalty = l2; C = 1; solver = lbfgs; maximum iteration number = 100;
- SVM: C = 1; kernel = rbf, probability = True;
- KNN: number of neighbors = 5; algorithm = auto; distance = Euclidian;
- RF: number of estimators = 100; criterion for split = Gini impurity; no max depth; max features = $\sqrt{number\;of\;features}$; using bootstrap.

Each ML algorithm was trained and evaluated for each individual feature (1 input); for the conventional features (9 inputs); for the proposed features (5 inputs); and for all the features (14 inputs). The summary of all 17 possible input options for each of the ML algorithms is given in Table 1. Each of the algorithm and input feature combinations was evaluated using a subject-wise leave-one-out cross-validation, where the trial data from each subject belonged to a single fold (30 folds in total), and in each iteration, one fold was used for testing and the remaining ones for training. The prediction value, label, and prediction probability for each instance of a test fold were saved and concatenated so that after the cross-validation was finished, the evaluation metrics (accuracy, ACC; sensitivity, Se; specificity, Sp; F1 score; area under the receiver operating characteristic curve, AUROC) could be calculated on the entirety of the test folds.

Other forms of ML evaluation, such as stratified 5-fold or stratified 3-fold subject-wise evaluations, were attempted (a single fold having 30/5 = 6 or 30/3 = 10 subjects, respectively) but showed a negligible difference in terms of the evaluation metrics when compared to the leave-one-out method.

Feature ranking was performed in order to sort the features in terms of their importance with regard to dyslexia classification, and it was based on the decrease in impurity in the RF algorithm [45]. The statistical analysis was then performed in the SPSS software (16.0, IBM Corp., New York, NY, USA) for each of the features that were shown to be indicative of dyslexic behavior by the feature ranking. First, the Mann–Whitney test was performed to compare the feature values for each color configuration separately between the two subject groups (dyslexic and control). Second, the Levene test of homogeneity of variances was performed with the goal of comparing the dispersity of the observed feature for each color configuration separately, between the two subject groups. The final part of the statistical analysis included a Wilcoxon signed ranks test performed within the dyslexic subject group, comparing the feature values between different color configurations. This analysis was performed for each pair of color configurations to determine whether a given color configuration was more favorable for the dyslexic subjects.

**Figure 2:**
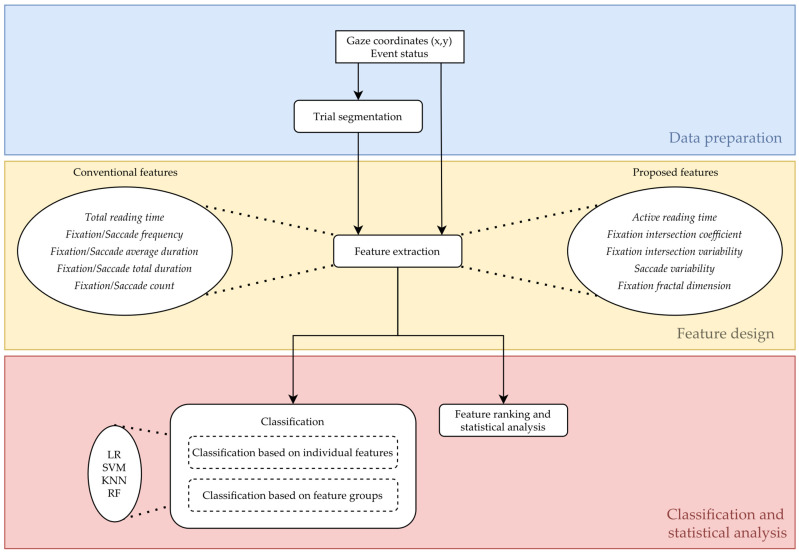
The analysis pipeline. LR—logistic regression; SVM—support vector machine; KNN—k-nearest neighbors; RF—random forest.

The analysis pipeline performed in this paper is given in Figure 2.

## 4. Results

The average metrics achieved on the test sets for the four ML algorithms (LR, SVM, KNN, RF), using three different feature sets (conventional, proposed, and all features) as inputs, are given in Table 2.

The achieved results show an overall high accuracy and a consistently better result when using the proposed features as well as the all features as inputs in comparison to the conventional ones. The best accuracy for both the proposed features as inputs and the all features as inputs was obtained by the LR algorithm, and it convincingly surpassed the best accuracy of 85% obtained for the conventional features by the SVM algorithm.

The average test set accuracy achieved when each individual feature is used as the ML input is shown in Table 3. The other metrics for single feature evaluation are presented in Appendix A.

The best accuracy was achieved for the *Fixation intersection variability* feature. The second and third best accuracies were achieved for the *Fixation intersection coefficient* and the *Fixation fractal dimension*. The accuracies achieved for these three features for all the ML algorithms were higher than the accuracies achieved when using all the conventional features as inputs.

**Figure 3:**
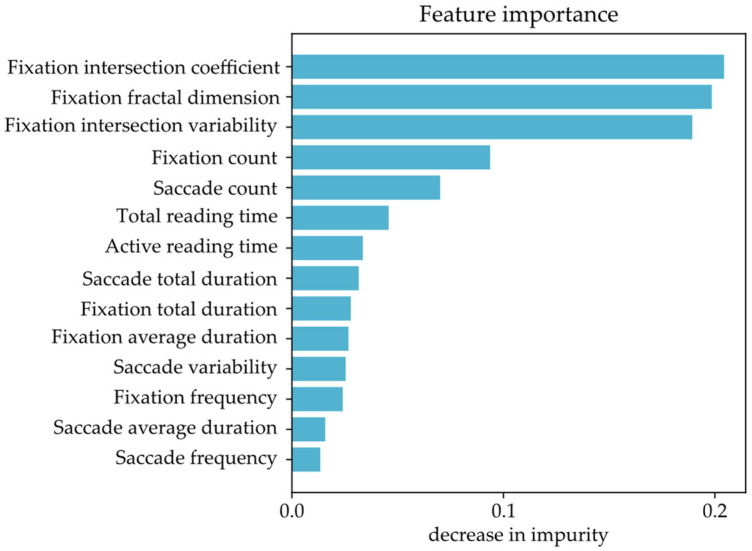
Feature importance of the eye-tracking features based on the decrease in the impurity of the random forest algorithm.

The importance of each individual feature was also ranked using the decrease in impurity in the RF algorithm [45], and the results are shown in Figure 3.

The feature importance ranking indicates that the three proposed spatial features (*Fixation intersection coefficient*, *Fixation fractal dimension*, and *Fixation intersection variability*) that achieved the highest individual accuracy do indeed contribute to a high classification accuracy when observed as part of a feature set. Considering this, the three proposed features were used for further statistical analysis.

**Figure 4:**
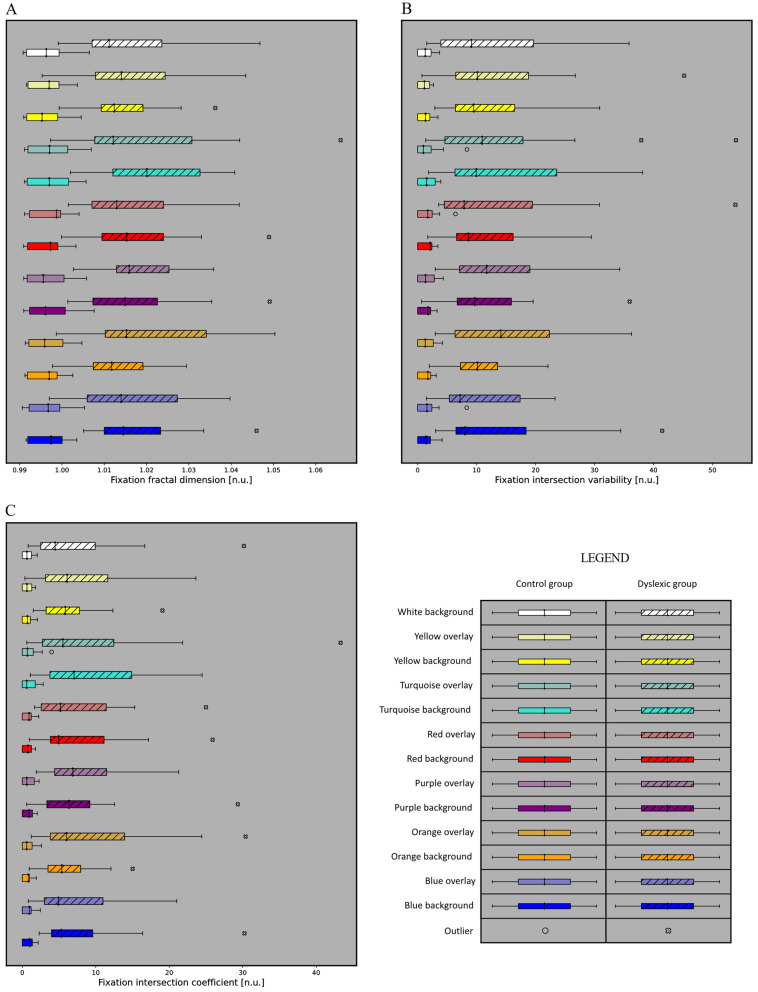
The boxplots of (**A**) *Fixation intersection coefficient*, (**B**) *Fixation fractal dimension*, (**C**) *Fixation intersection variability* for each color configuration and two subject groups (dyslexic and control).

The boxplots of the *Fixation intersection coefficient*, *Fixation fractal dimension*, and *Fixation intersection variability* for each color configuration and each subject group (dyslexic and control) are shown in Figure 4.

The boxplots show that there is a clear difference between the dyslexic and control classes for each color configuration (the control group has much lower feature values than the dyslexic group). This was further proved by the statistical analysis. For the three most important features, for each color configuration, a statistically significant difference was achieved between the subject classes (*p* < 0.001) using the Mann–Whitney test. Furthermore, the Levene test of the dispersity between the subject groups also showed a statistically significant difference for each of the three fixation complexity features (*Fixation intersection coefficient*, *Fixation fractal dimension*, *Fixation intersection variability*) for every color configuration (*p* < 0.01). The Mann–Whitney test shows that the feature values significantly differ between the groups, and the Levene test of dispersity shows that for each color configuration, the dyslexic group has many more dispersed data points than the control group.

**Figure 5:**
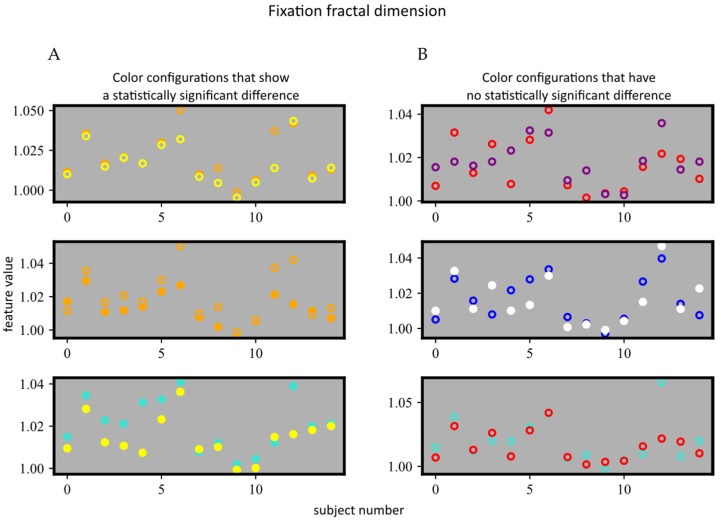
The visualization of data for all dyslexic subjects, for the three color configurations that (**A**) show a statistically significant difference and (**B**) show no statistical difference. Dots represent the background color configurations, and circles represent the overlay color configurations.

In order to determine whether there was a color that had a more positive influence on dyslexic subjects (the color that would produce the lowest feature values, as close as possible to the values of the control group), a statistical analysis was performed within the dyslexic subject group, comparing each pair of color configurations. The Wilcoxon signed ranks test showed that there was a statistically significant difference (*p* < 0.01) only for three pairs of color configurations and only for a single feature (*Fixation fractal dimension*): (1) yellow overlay and orange overlay, (2) orange background and yellow background, and (3) turquoise background and yellow background. The visualization of the configuration pairs for which there was a statistically significant difference, as well as for three arbitrary configurations for which there was no significant difference, is shown in Figure 5.

## 5. Discussion

In this paper, several ML algorithms and statistical tests were performed with the goal of analyzing the dyslexic tendencies in a group of 30 children (15 dyslexic and 15 control). The text was written in the subjects’ native language, Serbian, which has a perfect matching between letters and phonemes. Considering dyslexia detection in such languages (the ones with a shallow orthographic system) is often quite difficult; an accuracy of 94% achieved on the balanced dataset used in this paper (F1 score 0.93 and AUROC 0.96) (Table 2) shows a promising result that is comparable to the ones achieved in the literature [29,30,32,33,35,36,37,39,40,41] which were performed on languages with deeper orthographic systems. As the Serbian language has a shallow orthographic system, making dyslexia harder to diagnose, we consider the observed subject pool relevant for the performed research purposes for a language such as Serbian. Although the number of participants used in this study is lower than the subject groups found in the literature [28,29,36,39,40], the number of total used trials (378 trials, explained in Section 3.1) provided enough data for the performed type of machine learning analysis.

The three most important features (*Fixation intersection coefficient*, *Fixation fractal dimension*, and *Fixation intersection variability*, Figure 3) that describe the fixation gaze complexity achieved a decently high accuracy (89% or higher, Table 3), even when they were used as the single input feature for the ML algorithms. The importance of feature design and data interpretation has shown to be quite significant as a single spatial feature describing fixation gaze complexity achieved a better accuracy (91% for *Fixation intersection coefficient*) than all of the observed conventional features combined (85%). It is important to note that the fixation complexity features clearly have lower values for the control subjects and higher values for the dyslexic ones. The fixation complexity features, and consequently the gaze pattern complexity, could therefore be considered an indication of reading difficulties that can be observed in dyslexic subjects.

The proposed features should also be of use in dyslexia analysis for languages besides Serbian as struggling to focus on words could yield similar chaotic fixation movements in other languages. The drawback of the features is that they do require a certain sampling frequency and eye-tracker precision as the characterization of fixations that is used in this work does rely on detecting fine eye movements. The field of view of the reader can also influence the quality of the feature as reading from a further/shorter distance from the screen/paper could enable the reader to have a different number of words within a single focus point. This can, in turn, make the chaotic movement of the gaze either harder to detect or perhaps more saccadic, which might influence the separability of the classes.

The statistical analysis showed that the spatial features provide clear class separability regardless of color configuration, as seen in Figure 4. The statistical differences between the subject groups for all the color configurations show that a single color cannot be used to make reading easier, to the degree that the dyslexic and control groups are not separable.

The comparison between color configurations for dyslexic subjects shows that there could be color configurations that are more favorable than others. The analysis within the dyslexic group also showed a statistically significant difference only between three pairs of colors, as seen in Figure 5, indicating that none of the colors, universally, makes reading easier or harder when compared to the other ones. A lack of a consistently superior configuration, however, indicates that the colors have a different effect on each subject and that, in order to make reading easier for children with dyslexia, an individualistic approach would most likely be the best solution. The same conclusion could be reached by observing the statistical analysis between subject groups, as the statistical significance was prominent for each color configuration, indicating that none of the colors stands out in the sense of making dyslexic and control subjects more similar in their reading patterns.

## 6. Conclusions

The paper introduced a novel spatiotemporal feature set for recognition of gaze patterns in dyslexic native Serbian speakers. The proposed feature set has shown a significant classification improvement in comparison to conventional eye-tracking features (94% vs. 85%). The statistical analysis between subject classes (dyslexic and control) found high class separability, independent of color configuration. A statistical analysis related to the color impact on reading performance was accomplished within the dyslexic subject group and showed high inter-subject variability.

The performed study was limited by the number of participants and by the usage of a high-precision eye tracker. However, the obtained results are promising in the field of dyslexia detection, and further work could include an introduction of the features measured from other sensor systems (including low-cost systems), analyzing a larger number of subjects, or a subject base of broader age distribution. Analyzing the data from different eye trackers and combining the obtained dataset with other datasets (possibly in different languages) would also be of interest for future work.

## Figures and Tables

**Table 1:** ML algorithm input feature options.

Algorithm Input Options			
No.	Feature Set Input Options	No.	Single Feature Input *(1 Input)*
1.	Conventional features *(9 inputs)*: *Fixation count, Fixation total duration, Fixation frequency, Fixation average duration, Saccade count, Saccade total duration, Saccade frequency, Saccade average duration, Total reading time*	4.	*Active reading time*
		5.	*Fixation intersection coefficient*
		6.	*Saccade variability*
		7.	*Fixation intersection variability*
2.	Proposed features *(5 inputs)*: *Active reading time, Fixation intersection coefficient, Saccade variability, Fixation intersection variability, Fixation fractal dimension*	8.	*Fixation fractal dimension*
		9.	*Fixation count*
		10.	*Fixation total duration*
		11.	*Fixation frequency*
		12.	*Fixation average duration*
		13.	*Saccade count*
		14.	*Saccade total duration*
		15.	*Saccade frequency*
3.	Conventional and Proposed features *(14 inputs)*	16.	*Saccade average duration*
		17.	*Total reading time*

**Table 2:** Feature group classification evaluation metrics (the proposed feature results marked with bold text). ACC—accuracy; Se—sensitivity; Sp—specificity; AUROC—area under the receiver operating characteristic curve.

Feature Group	ML Algorithm			
	LR	SVM	KNN	RF
Conventional features				
ACC	0.84	0.85	0.81	0.82
Se	0.78	0.72	0.66	0.75
Sp	0.90	0.97	0.94	0.92
F1 score	0.83	0.82	0.77	0.81
AUROC	0.88	0.89	0.87	0.86
**Proposed features**				
ACC	**0.94**	**0.93**	**0.88**	**0.93**
Se	**0.89**	**0.88**	**0.78**	**0.89**
Sp	**0.98**	**0.98**	**0.98**	**0.97**
F1 score	**0.93**	**0.93**	**0.86**	**0.93**
AUROC	**0.96**	**0.98**	**0.94**	**0.95**
All features				
ACC	0.94	0.93	0.87	0.94
Se	0.89	0.87	0.75	0.86
Sp	0.98	0.98	0.98	0.97
F1 score	0.93	0.92	0.84	0.91
AUROC	0.96	0.97	0.94	0.94

**Table 3:** Classification accuracies for single feature inputs.

	Feature	ML Algorithm			
		SVM	LR	RF	KNN
*Proposed*	*Active reading time*	0.78	0.75	0.74	0.76
	*Fixation intersection coefficient*	0.90	0.90	0.89	0.89
	*Saccade variability*	0.74	0.74	0.76	0.73
	***Fixation intersection variability***	**0.91**	**0.90**	**0.91**	**0.91**
	*Fixation fractal dimension*	0.89	0.90	0.89	0.89
*Conventional*	*Fixation count*	0.84	0.85	0.84	0.84
	*Fixation total duration*	0.78	0.74	0.77	0.76
	*Fixation frequency*	0.35	0.30	0.52	0.63
	*Fixation average duration*	0.46	0.49	0.48	0.63
	*Saccade count*	0.81	0.81	0.83	0.82
	*Saccade total duration*	0.78	0.74	0.76	0.76
	*Saccade frequency*	0.57	0.47	0.63	0.57
	*Saccade average duration*	0.48	0.56	0.60	0.56
	*Total reading time*	0.80	0.77	0.74	0.75

## Supplementary Materials

The following supporting information can be downloaded at: https://www.mdpi.com/article/10.3390/s22134900/s1, Figure S1: White background test board example; Figure S2: Yellow background test board example; Figure S3: Red overlay test board example; Figure S4: Orange background test board example; Figure S5: Yellow overlay test board example; Figure S6: Orange overlay test board example; Figure S7: Blue overlay test board example; Figure S8: Purple background test board example; Figure S9: Purple overlay test board example; Figure S10: Red background test board example; Figure S11: Turquoise overlay test board example; Figure S12: Blue background test board example; Figure S13: Turquoise background test board example.

## Appendix A

**Table A1:** Sensitivity for single feature inputs.

	Feature	ML Algorithm			
		SVM	LR	RF	KNN
*Proposed*	*Active reading time*	0.59	0.65	0.60	0.61
	*Fixation intersection coefficient*	0.85	0.84	0.83	0.84
	*Saccade variability*	0.54	0.61	0.62	0.58
	***Fixation intersection variability***	**0.85**	**0.82**	**0.85**	**0.85**
	*Fixation fractal dimension*	0.84	0.87	0.83	0.84
*Conventional*	*Fixation count*	0.74	0.78	0.76	0.77
	*Fixation total duration*	0.59	0.64	0.60	0.59
	*Fixation frequency*	0.31	0.29	0.48	0.50
	*Fixation average duration*	0.32	0.33	0.43	0.50
	*Saccade count*	0.74	0.75	0.82	0.83
	*Saccade total duration*	0.59	0.64	0.61	0.59
	*Saccade frequency*	0.44	0.46	0.42	0.45
	*Saccade average duration*	0.28	0.46	0.44	0.44
	*Total reading time*	0.61	0.65	0.59	0.62

**Table A2:** Specificity for single feature inputs.

	Feature	ML Algorithm			
		SVM	LR	RF	KNN
*Proposed*	*Active reading time*	0.95	0.83	0.89	0.90
	*Fixation intersection coefficient*	0.95	0.96	0.93	0.94
	*Saccade variability*	0.94	0.85	0.87	0.86
	***Fixation intersection variability***	**0.96**	**0.97**	**0.94**	**0.96**
	*Fixation fractal dimension*	0.93	0.92	0.93	0.93
*Conventional*	*Fixation count*	0.93	0.91	0.91	0.91
	*Fixation total duration*	0.95	0.83	0.92	0.91
	*Fixation frequency*	0.39	0.34	0.59	0.76
	*Fixation average duration*	0.60	0.64	0.60	0.77
	*Saccade count*	0.87	0.85	0.82	0.83
	*Saccade total duration*	0.95	0.83	0.89	0.91
	*Saccade frequency*	0.70	0.47	0.73	0.70
	*Saccade average duration*	0.69	0.66	0.81	0.70
	*Total reading time*	0.97	0.86	0.83	0.87

**Table A3:** F1 score for single feature inputs.

	Feature	ML Algorithm			
		SVM	LR	RF	KNN
*Proposed*	*Active reading time*	0.72	0.71	0.70	0.71
	*Fixation intersection coefficient*	0.89	0.89	0.87	0.88
	*Saccade variability*	0.67	0.69	0.70	0.68
	***Fixation intersection variability***	**0.90**	**0.89**	**0.89**	**0.90**
	*Fixation fractal dimension*	0.88	0.89	0.87	0.88
*Conventional*	*Fixation count*	0.81	0.83	0.82	0.83
	*Fixation total duration*	0.72	0.71	0.71	0.70
	*Fixation frequency*	0.32	0.28	0.50	0.57
	*Fixation average duration*	0.37	0.39	0.47	0.58
	*Saccade count*	0.78	0.79	0.82	0.83
	*Saccade total duration*	0.72	0.71	0.71	0.70
	*Saccade frequency*	0.50	0.46	0.50	0.51
	*Saccade average duration*	0.34	0.51	0.53	0.50
	*Total reading time*	0.74	0.73	0.67	0.71

**Table A4:** Area under the receiver operating characteristic curve for single feature inputs.

	Feature	ML Algorithm			
		SVM	LR	RF	KNN
*Proposed*	*Active reading time*	0.67	0.79	0.73	0.75
	*Fixation intersection coefficient*	0.94	0.95	0.92	0.94
	*Saccade variability*	0.74	0.73	0.77	0.77
	***Fixation intersection variability***	**0.94**	**0.95**	**0.93**	**0.94**
	*Fixation fractal dimension*	0.93	0.96	0.92	0.94
*Conventional*	*Fixation count*	0.87	0.89	0.86	0.87
	*Fixation total duration*	0.67	0.78	0.72	0.72
	*Fixation frequency*	0.37	0.32	0.59	0.61
	*Fixation average duration*	0.51	0.38	0.58	0.62
	*Saccade count*	0.85	0.87	0.85	0.87
	*Saccade total duration*	0.67	0.78	0.72	0.72
	*Saccade frequency*	0.53	0.34	0.59	0.58
	*Saccade average duration*	0.40	0.54	0.60	0.58
	*Total reading time*	0.68	0.79	0.73	0.76
