# Supplementary figures and images for: Spatiotemporal Eye-Tracking Feature Set for Improved Recognition of Dyslexic Reading Patterns in Children
Source: Sensors (Basel). 2022 Jun 29;22(13):4900. doi: 10.3390/s22134900 (PMC9269601; doi:10.3390/s22134900)

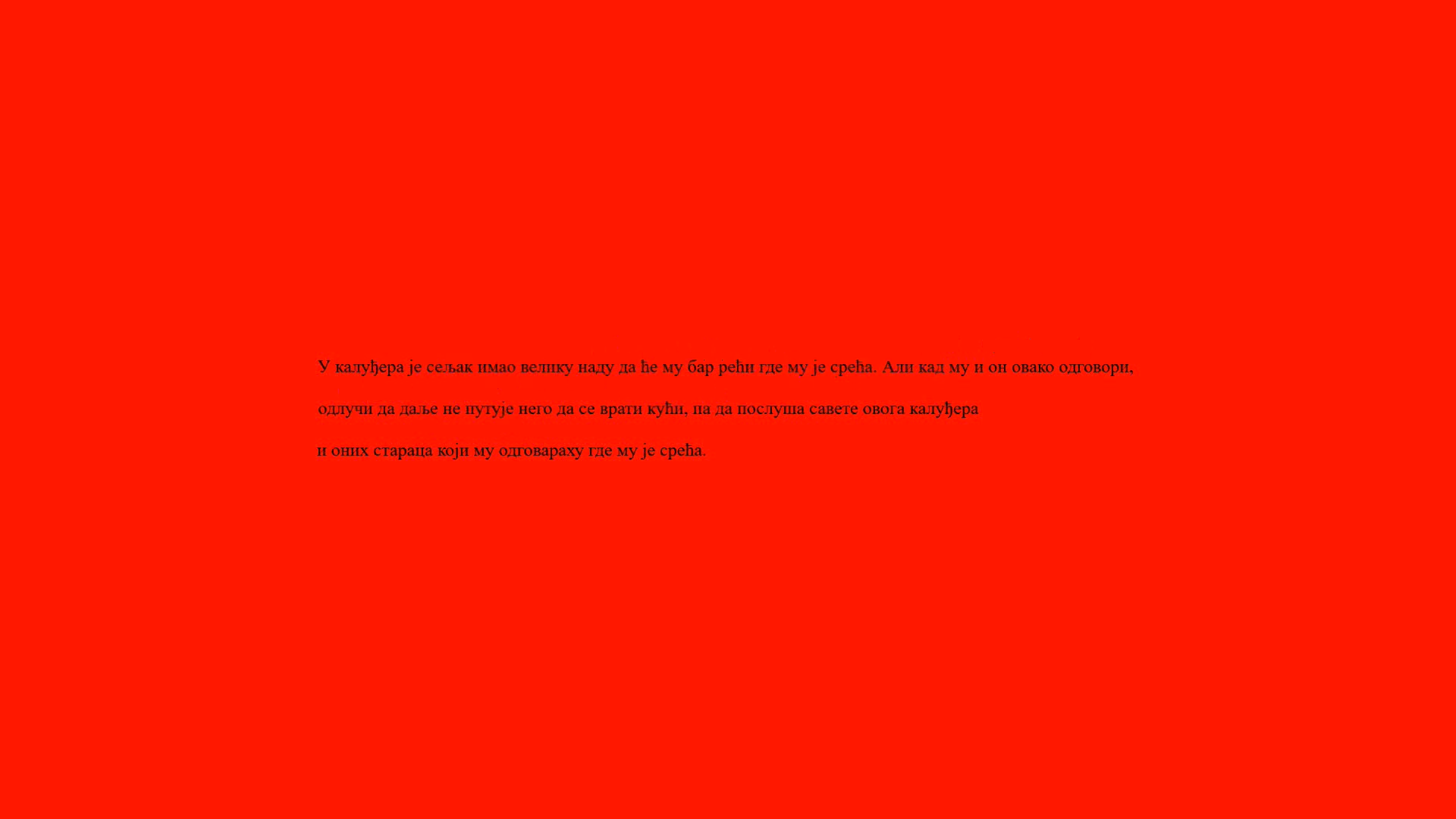

Supplement: Supplementary file 1 [file sensors-22-04900-s001.zip › Figure S10_Red_background.png]

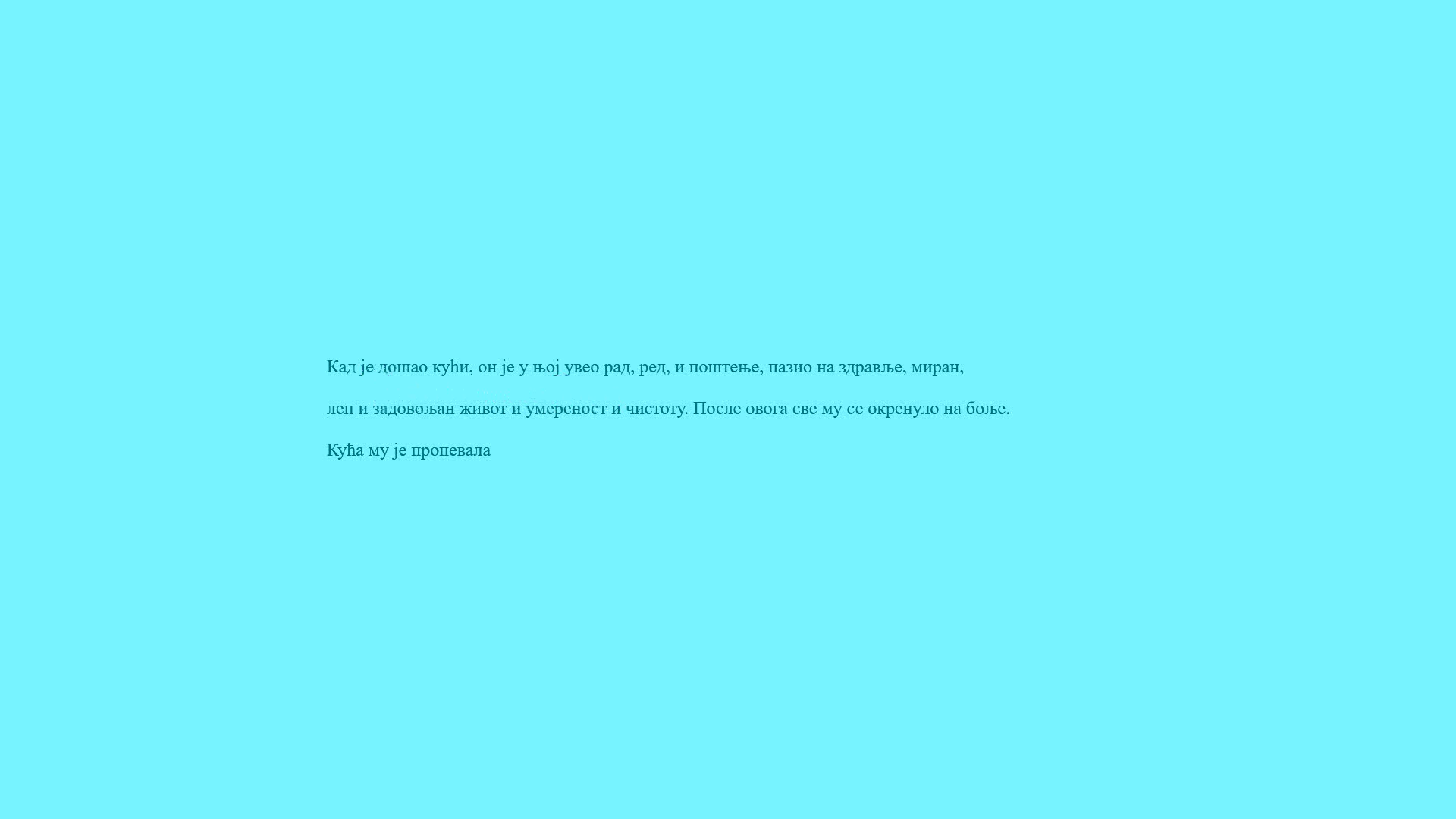

Supplement: Supplementary file 1 [file sensors-22-04900-s001.zip › Figure S11_Turquoise_overlay.png]

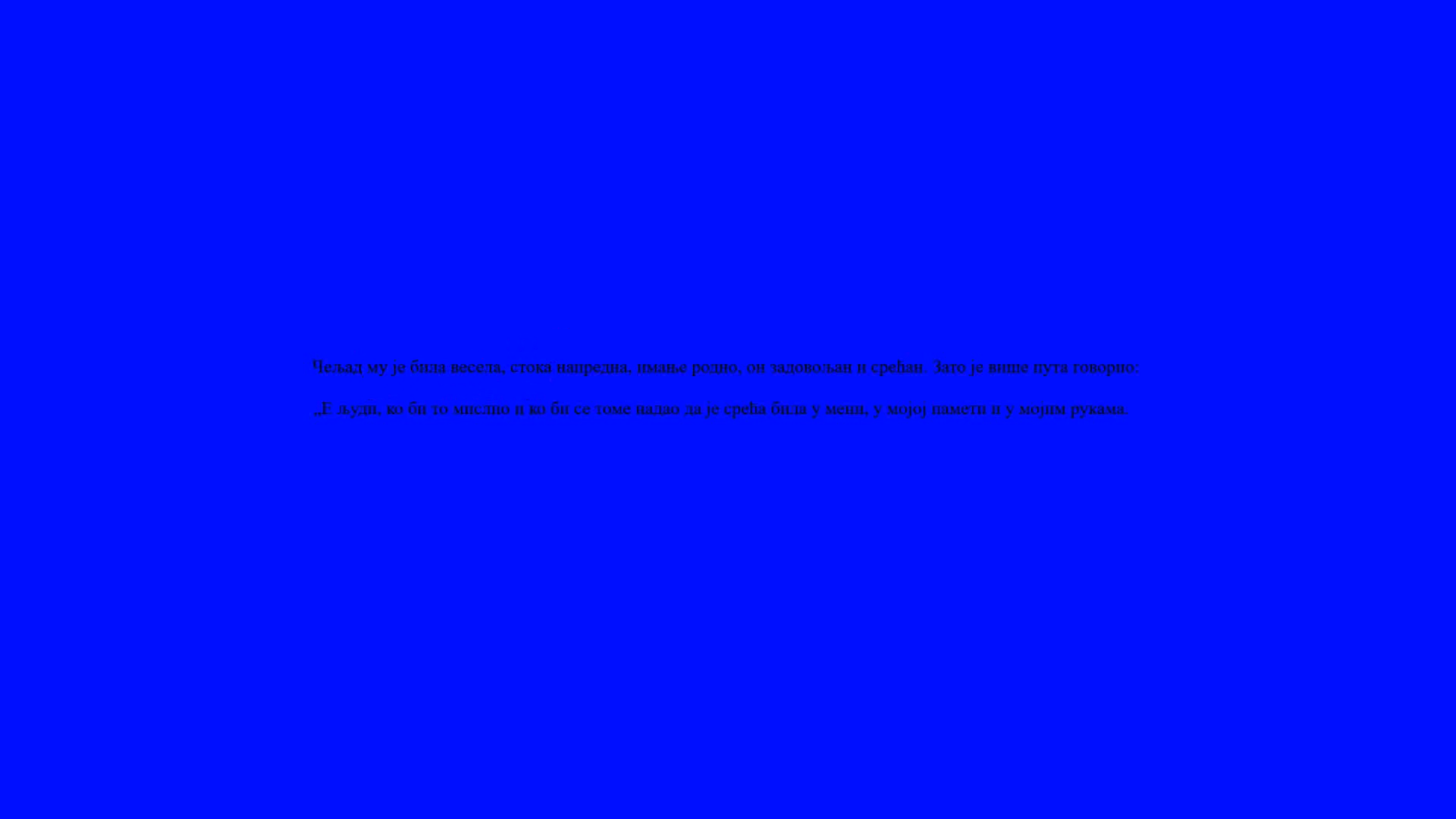

Supplement: Supplementary file 1 [file sensors-22-04900-s001.zip › Figure S12_Blue_background.png]

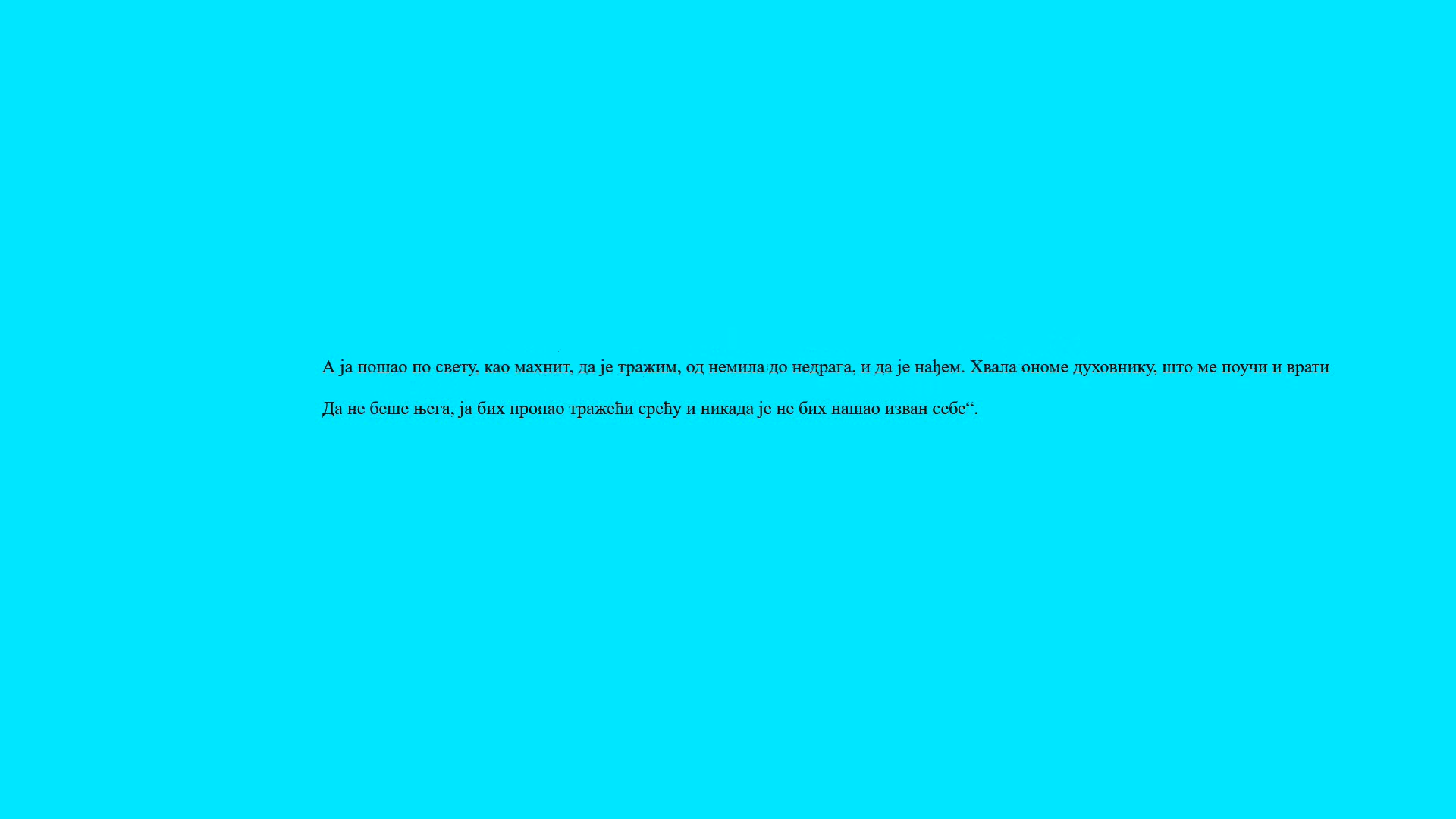

Supplement: Supplementary file 1 [file sensors-22-04900-s001.zip › Figure S13_Turqoise_background.png]

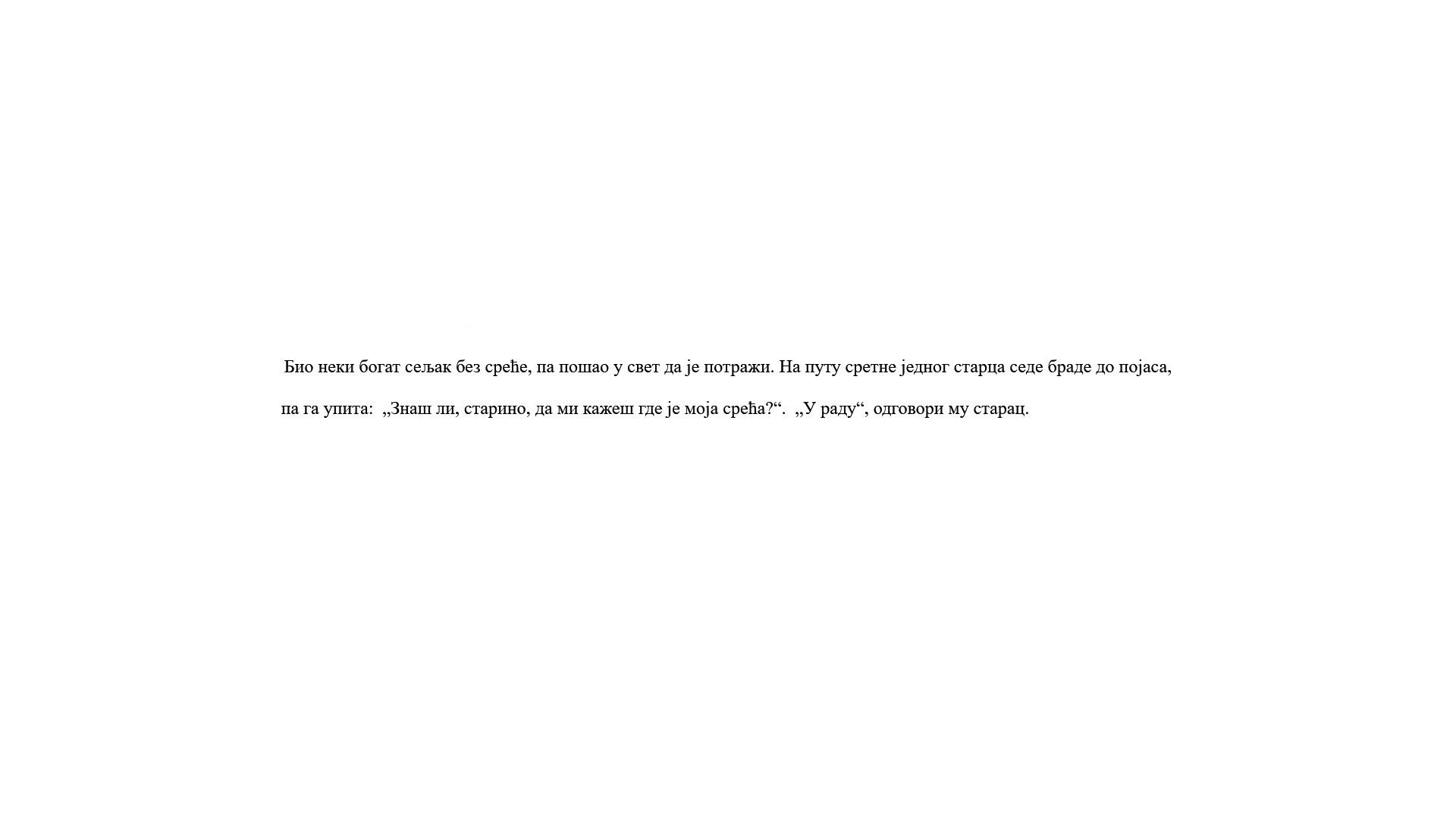

Supplement: Supplementary file 1 [file sensors-22-04900-s001.zip › Figure S1_White_background.png]

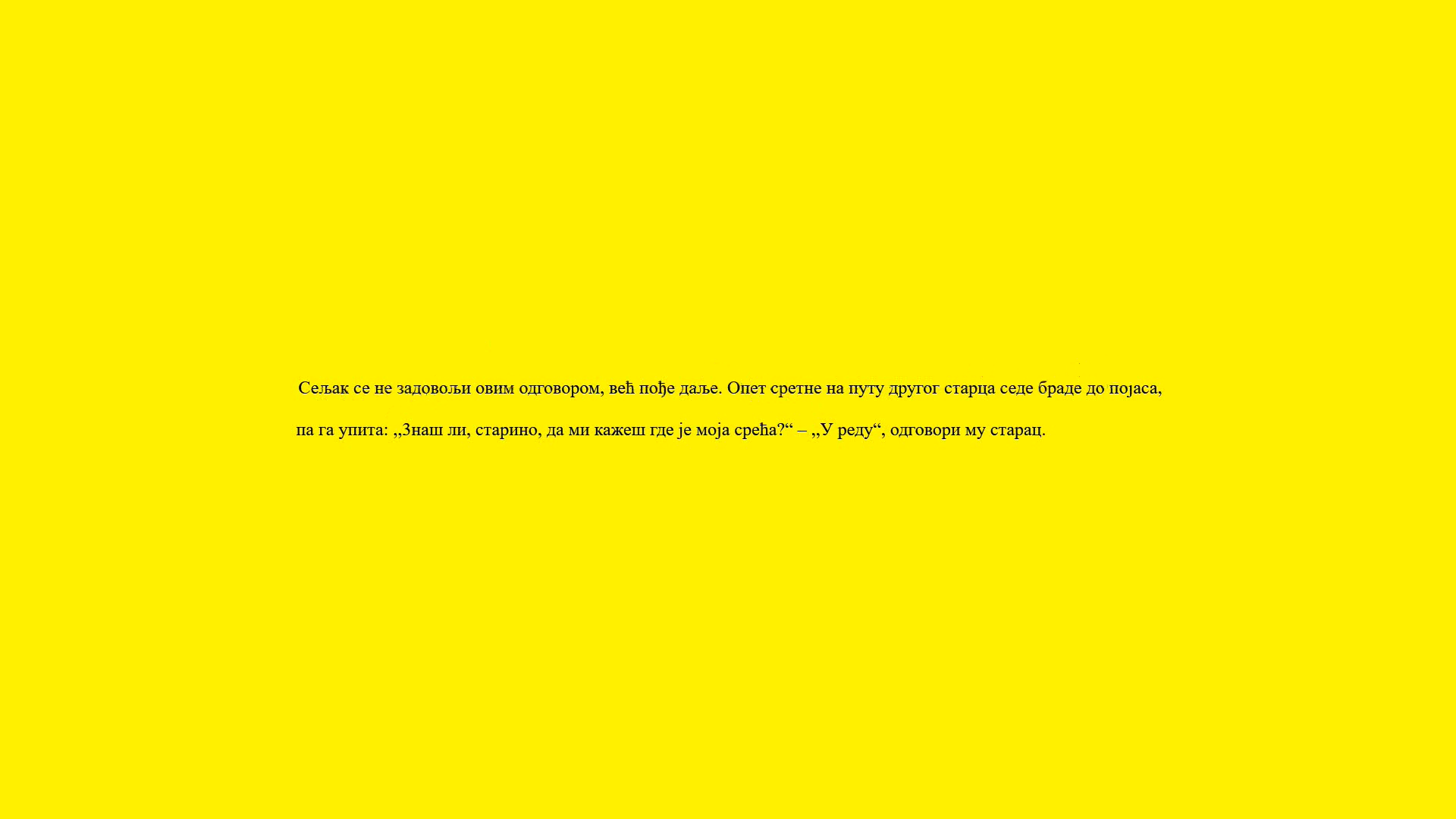

Supplement: Supplementary file 1 [file sensors-22-04900-s001.zip › Figure S2_Yellow_background.png]

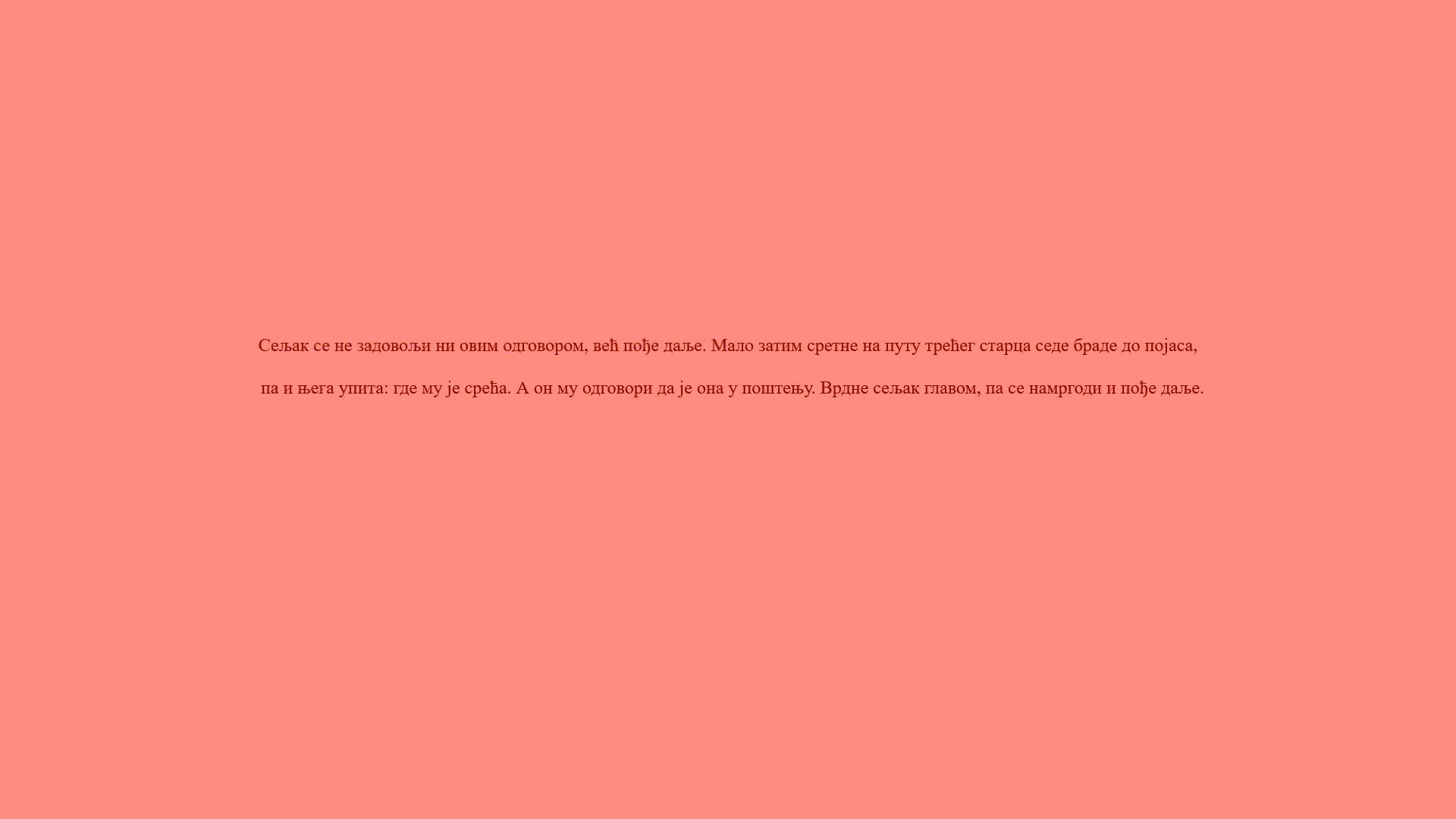

Supplement: Supplementary file 1 [file sensors-22-04900-s001.zip › Figure S3_Red_overlay.png]

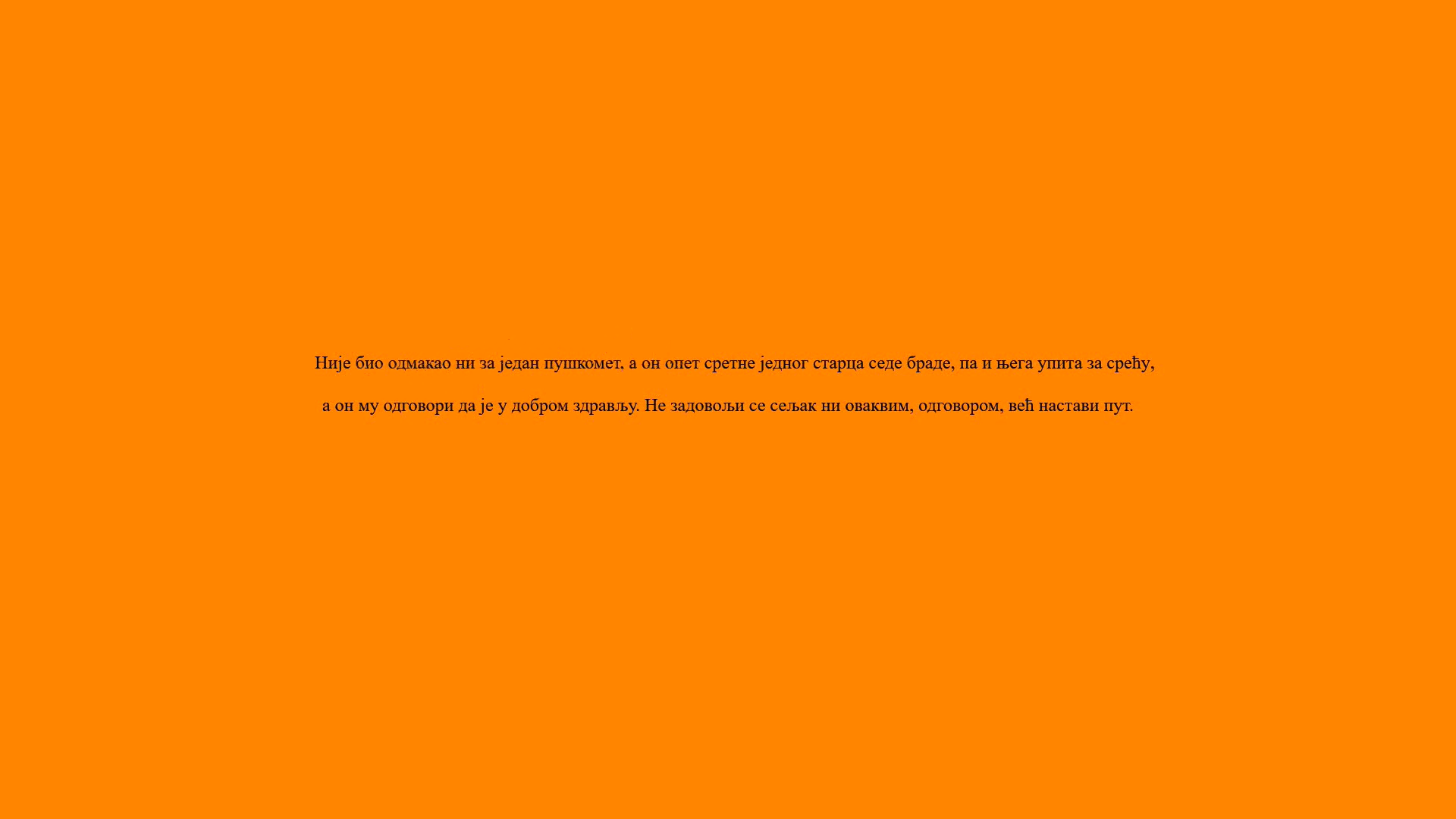

Supplement: Supplementary file 1 [file sensors-22-04900-s001.zip › Figure S4_Orange_background.png]

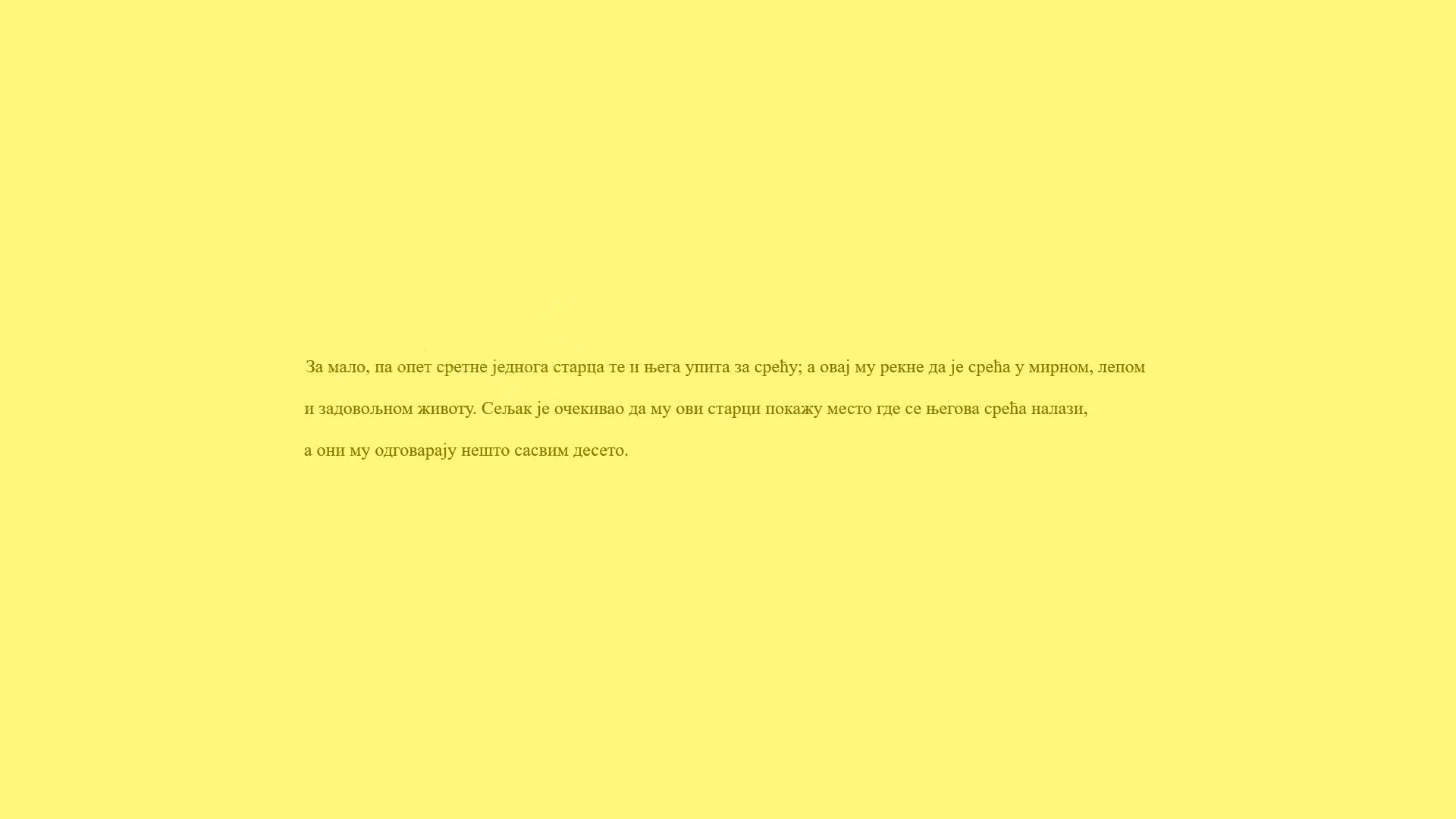

Supplement: Supplementary file 1 [file sensors-22-04900-s001.zip › Figure S5_Yellow_overlay.png]

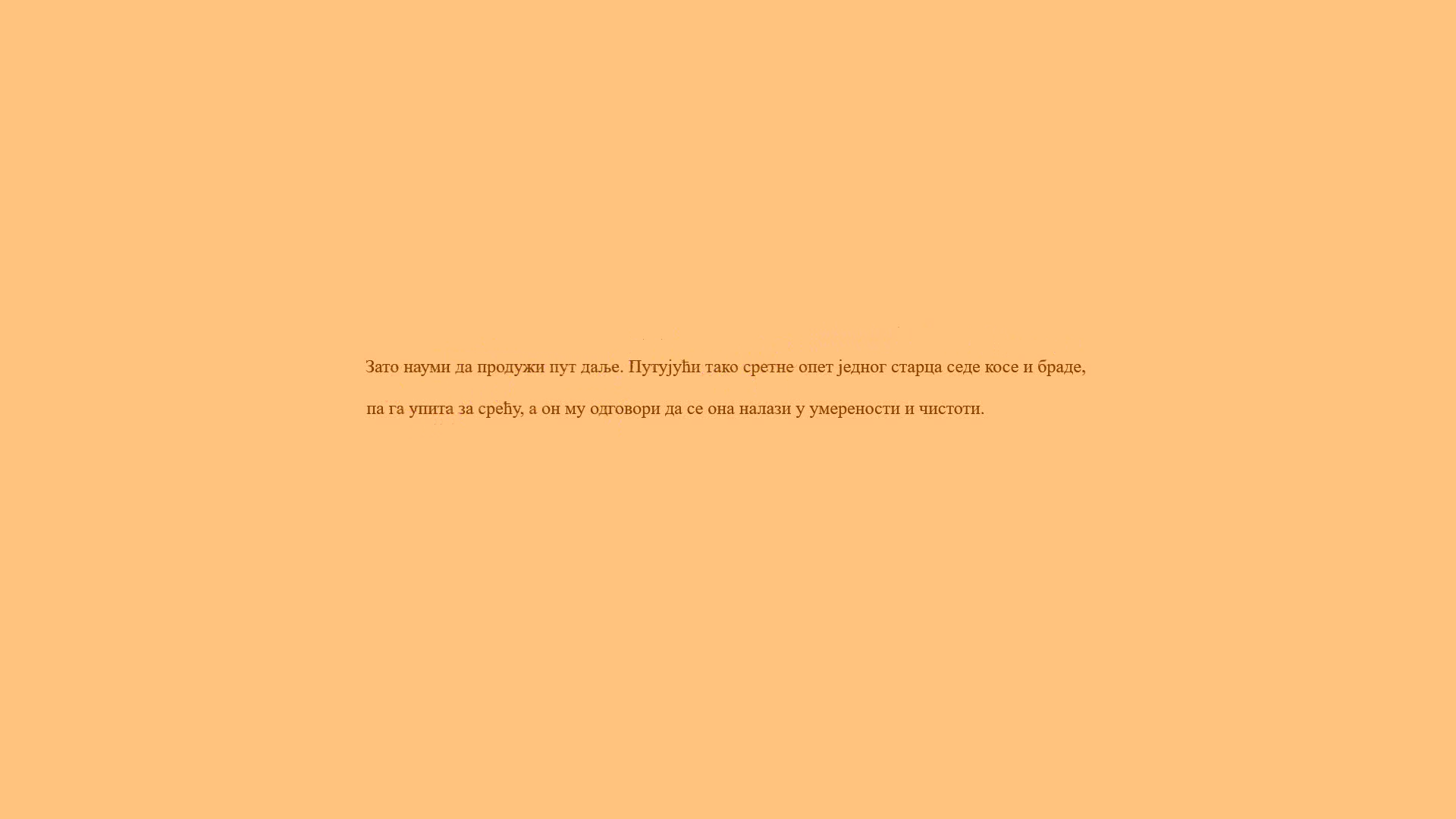

Supplement: Supplementary file 1 [file sensors-22-04900-s001.zip › Figure S6_Orange_overlay.png]

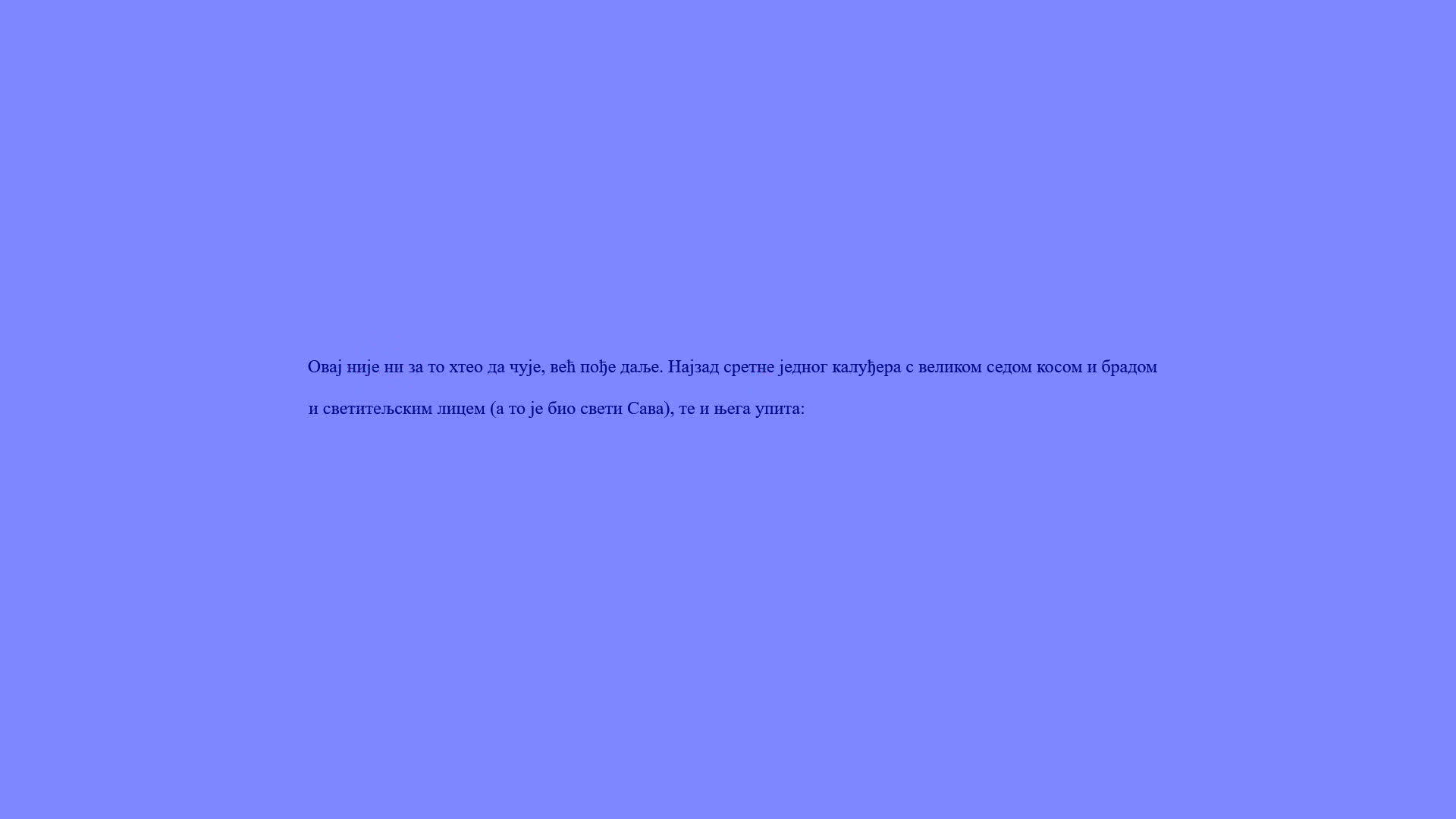

Supplement: Supplementary file 1 [file sensors-22-04900-s001.zip › Figure S7_Blue_overlay.png]

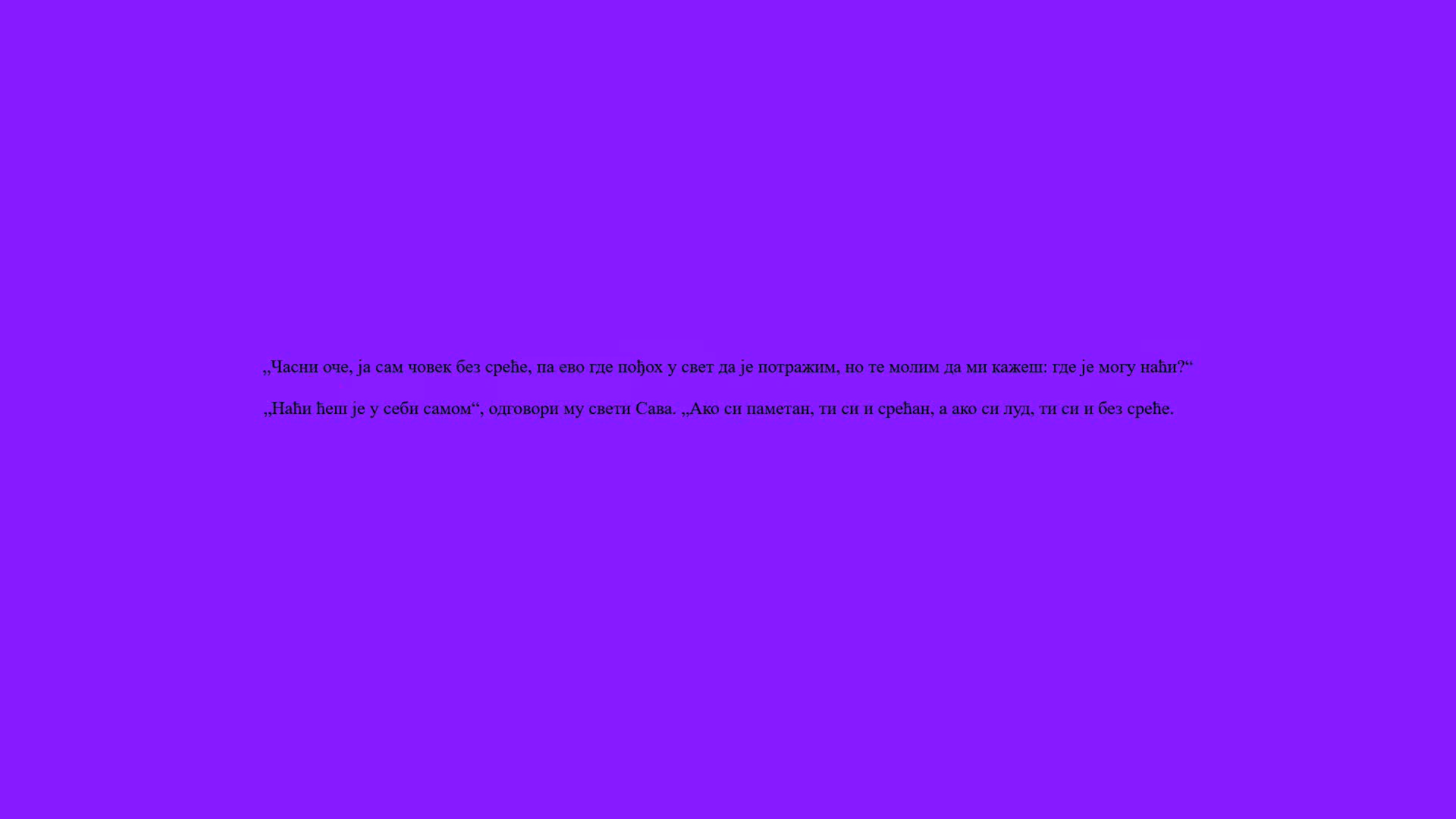

Supplement: Supplementary file 1 [file sensors-22-04900-s001.zip › Figure S8_Purple_background.png]

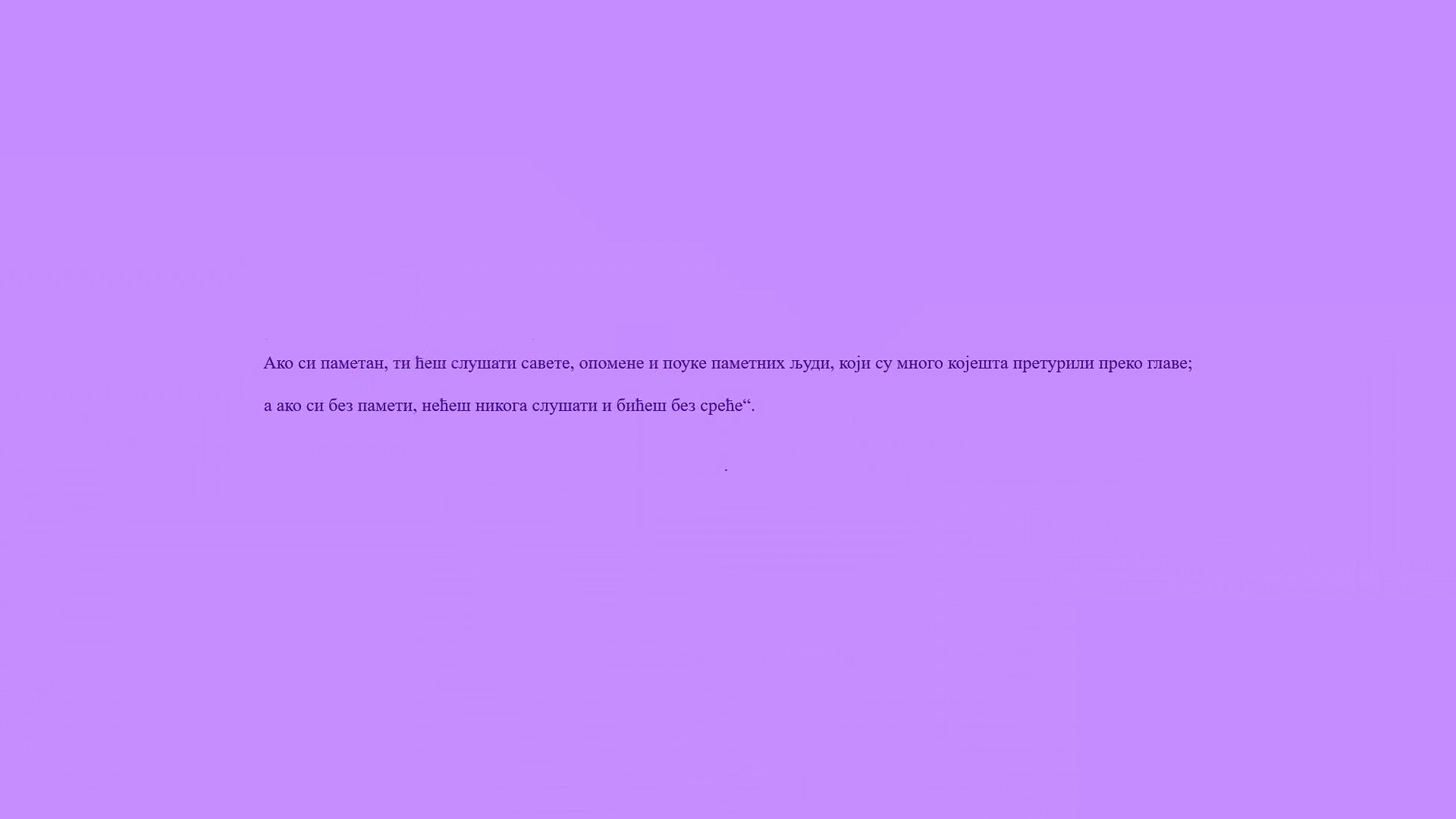

Supplement: Supplementary file 1 [file sensors-22-04900-s001.zip › Figure S9_Purple_overlay.png]
